# Validating SPICES as a Screening Tool for Frailty Risks among Hospitalized Older Adults

**DOI:** 10.1155/2014/846759

**Published:** 2014-04-27

**Authors:** Harriet Udin Aronow, Jeff Borenstein, Flora Haus, Glenn D. Braunstein, Linda Burnes Bolton

**Affiliations:** Cedars-Sinai Medical Center, 8700 Beverly Boulevard, Los Angeles, CA 90048, USA

## Abstract

Older patients are vulnerable to adverse hospital events related to frailty. SPICES, a common screening protocol to identify risk factors in older patients, alerts nurses to initiate care plans to reduce the probability of patient harm. However, there is little published validating the association between SPICES and measures of frailty and adverse outcomes. This paper used data from a prospective cohort study on frailty among 174 older adult inpatients to validate SPICES. Almost all patients met one or more SPICES criteria. The sum of SPICES was significantly correlated with age and other well-validated assessments for vulnerability, comorbid conditions, and depression. Individuals meeting two or more SPICES criteria had a risk of adverse hospital events three times greater than individuals with either no or one criterion. Results suggest that as a screening tool used within 24 hours of admission, SPICES is both valid and predictive of adverse events.

## 1. Introduction

Older adult inpatients admitted to acute hospital are particularly vulnerable to adverse hospital events associated with factors related to frailty [1, 2]. Early identification of vulnerability and appropriate clinical intervention are essential to the prevention of hospital acquired adverse events [3]. The patient care team is responsible for maintaining the health and safety of the whole patient based on assessed patient conditions and monitoring responses to treatment.

SPICES is an acronym for a brief protocol for multidimensional assessment to identify risk factors related to caring for older adults: skin integrity; problems eating; incontinence;** c**onfusion; evidence of falls; and sleep disturbance [4]. The simple-to-use screening strategy alerts the bedside nurse to be vigilant in the surveillance of patients and to initiate care team activities. The patient care team goals are to reduce the probability of patient harm related to geriatric syndromes, whether an identified syndrome is directly related to the reason for admission or not. SPICES is commonly used and recommended by Nurses Improving Care for Healthsystem Elders (NICHE) as a valuable screening risk identification process. There is a paucity of data in the peer-reviewed literature that validates the SPICES criteria either based on an association with other measures of vulnerability or based on adverse outcomes in hospitalized older adults [5]. The purpose of this paper is to explore the association of SPICES clinical assessment criteria with adverse outcomes among a sample of older hospitalized patients who were included in a prospective cohort study of risk factors and hospital outcomes [6].

## 2. Methods and Materials

### 2.1. Overview

As part of a hospital-wide effort to improve efficiency and effectiveness of care, an interprofessional workgroup was assembled to focus on the quality and safety of patient care for frail older adults. Prior to designing an intervention, the group conducted a prospective cohort study of older adults admitted during the month of September, 2010. The sample consisted of 214 Medicare patients. The study aimed to (1) demonstrate the relevance, nature, and scope of potential quality improvement efforts within this population, (2) develop an interprofessional risk assessment tool for patients with a frailty phenotype early in admission, and (3) characterize gaps in processes and timeliness of frailty care and the impact on patient safety and clinical outcomes. The QI effort and the results of the prospective cohort study are reported elsewhere [6].

### 2.2. Secondary Source Data

The data from the prospective cohort study were used secondarily for the current study of SPICES. We focused on the subsample of 174 patients aged 65 or older. SPICES is typically scored by the registered nurse assigned to the patient or by a specially trained geriatric resource nurse [7]. SPICES criteria could be identified and coded from the wide array of patient data that were coded into the study data base. For the current validation study, we scored SPICES, in part, from documented assessments made by the registered nurses conducting usual admission assessments and, in part, from assessments completed by the research nurses conducting the prospective cohort study. Other validated risk screening assessments, not used to score SPICES, and subsequent patient outcomes were then used to validate the SPICES criteria.  Table 1 describes the coding logic used in the current study for each of the SPICES elements.

### 2.3. Validity Measures

To establish the validity of the SPICES assessment, it was necessary to have other measures of vulnerability with known psychometric properties that are related to risk for adverse outcomes. In our prospective cohort study we collected assessments that were then used secondarily to score SPICES and other assessments that were not used to score SPICES. The current validation analyses used only those scales and assessments that were not included in the scoring of SPICES.

Two scales collected concurrently, but not used to calculate SPICES, were used to establish criterion validity. The Vulnerable Elders Survey-13 (VES-13) is a survey, validated among community-residing older adults, that has been shown to identify at-risk populations in ambulatory and hospital settings [11–13]. VES-13 was administered by the research nurses in the prospective cohort study within the first day following the day of admission. Charlson Comorbidity Index (CI) is an assessment of the burden of acute and chronic disease that has been validated as a predictor of one-year mortality [14, 15]. The Charlson CI was rated by physicians from emergency department and admission documentation and measured within 24 hours of admission. We anticipated that the total positive SPICES elements would be significantly positively related to VES-13 and Charlson CI.

Construct validity was tested with other measures. Just as advancing age is generally associated with decreasing independence in function of activities of daily activity, we anticipated that SPICES would be positively associated with age. Burden of chronic disease is also associated with depression [16]. The Patient Health Questionnaire-2 (PHQ-2), a well-established depression screening assessment [17], was rated concurrently with initial study measures but was not included in SPICES. We expected that there would be a positive association of total SPICES+ with PHQ-2. PHQ-2 was administered by the research nurses in the first data collection within the first day following admission. The prospective cohort study data also included whether the patient was admitted from a skilled nursing facility; whether there was a recent prior hospital admission (within 30 days and two or more within six months); whether the patient had four or more active comorbidities with one being not controlled at the time of admission; and whether the patient had seven or more prescription medications at the time of admission. These measures were expected to be positively related to the patient's SPICES score. Finally, we did not anticipate that gender would be related to SPICES except through the mechanism of age (more women are among the older age group) or women's increased likelihood of having functional impairment.

Predictive validity was evaluated by examining the relationship of SPICES screening scores to outcome variations consistent with greater vulnerability. In the original prospective cohort study protocol, after the admission assessment, patients were followed to document adverse drug reactions, procedural complications, hospital-acquired infections, falls, the development of hospital acquired pressure ulcers, transfers to higher levels of care (e.g., from general medical surgical units to intensive care units), death, total hospital length of stay of one week or longer, and readmission within 30 days after discharge.

### 2.4. Analyses

The Statistical Package for Social Sciences (SPSS) was used for all analyses. The number of SPICES criteria identified as positive was tested with concurrent and outcome measures using simple chi-square and nonparametric spearman Rho correlation statistics. The use of SPICES as a screening tool was evaluated using a SPICES cut-off criterion and examining the relative risk of cooccurrence of other risk factors and adverse hospital outcomes.

## 3. Results and Discussion

### 3.1. Demographics

The patients included in this analysis (*N* = 174) comprised a convenience sample drawn from all medical and surgical admissions during a single month. All were insured through Medicare and aged 65 or older. Average age was 79.6 years (SD = 8.9; median = 80 years; range = 65−100), and 58.6% of the sample were women.

### 3.2. SPICES

The number and percent of the sample with positive results on each SPICES element are reported in Table 1. Most common was evidence of falls, with two-thirds of the sample screened positive on the Morse Falls Risk Scale. Least common was sleep disturbance with just four (2.3%) of the sample screening positive. All but 20 patients (11.5%) met at least one SPICES criterion. The range of total SPICES identified for each patient was 0–6. The distribution of total SPICES is shown in Table 2.

### 3.3. Association of SPICES with Criterion and Other Concurrent Measures

Total SPICES was significantly correlated with the criterion variables, VES-13 total score (*N* = 130, Rho = 0.559, *P* < 0.001) and Charlson Comorbidity Index (*N* = 174, Rho = 0.250, *P* = 0.001). SPICES was also significantly correlated with the construct measures of age (*N* = 174, Rho = 0.347, *P* < 0.001) and depression as measured by the PHQ-2 (*N* = 143, Rho = 0.259, *P* = 0.002).

Among this acute hospital patient sample, only 134 patients (77.0%) were able to respond to the VES-13 questions. Of the 40 patients with missing VES-13 data, 29 (72.5%) had four or more positive SPICES criteria and 37 (92.5%) met two or more of the SPICES criteria. Among the 31 patients who did not complete the PHQ-2 depression screen, 27 (87.1%) had four or more positive SPICES criteria and 29 (93.5%) had two or more positive SPICES.

### 3.4. SPICES for Screening

For the remainder of analyses, to establish the validity of SPICES as a screening criterion for vulnerability and to examine the association of SPICES to relatively rare adverse hospital outcomes, we dichotomized the distribution 0-1 versus 2 or more SPICES positive (SPICES2+). This split placed 37.4% of the sample in the lower risk category and 62.6% in the higher risk category.

Table 3 presents results of the association of the dichotomized SPICES2+ assessment with criterion, construct, and outcome measures. The likelihood of the association of two or more SPICES positive criteria with validation measures is shown by the relative risk of the validation measure being associated with the higher SPICES score.

There was strong agreement of risk as identified by SPICES2+ with the VES-13 criterion score for vulnerability (OR = 11.08, 95% CI = 4.542, 27.013, *P* < 0.001) and by both risk level cut-off criteria on the Charlson CI (see Table 3). From the set of other concurrent measures of frailty or vulnerability, all but prior hospitalizations were significantly associated with the SPICES2+ screening assessment. Female gender was associated with SPICES2+ (*P* = 0.058), but gender was nonsignificant when included in a multivariate regression with age ≥80 years and VES-13 frail.

Individual hospital adverse outcomes were uncommon to rare events (see Table 3). For example, three patients in the sample died during their admission. All three were screened “at risk” by SPICES2+, but the *n* was too small to establish a stable risk statistic. Most hospital adverse outcomes were not significantly associated with screening SPICES. SPICES2+ was associated with over four times greater likelihood of hospital length of stay of a week or more (OR = 4.642, 95% CI = 2.017, 10.686, *P* < 0.001). A positive finding on SPICES2+ was significantly associated with the occurrence of any one or more of adverse outcomes of hospitalization (OR = 3.04, 95% CI = 1.527, 6.054, *P* = 0.001). SPICES2+ was not associated with subsequent readmission within 30 days, with approximately 20% of each group readmitted within 30 days.

Definitions of vulnerability in older adult patients vary widely both in conceptualization and application [18, 19]. Development and operationalization of an evidence-based risk assessment tool for clinical practice are an important undertaking. SPICES is a commonly used screening protocol. This study examined whether SPICES was valid, when compared with other established scales and evidence-based frailty and vulnerability criteria, as a method for targeting a subgroup of patients at high risk for adverse hospital outcomes. We found that the SPICES screening criteria, using data collected by registered nurses available within 24 hours of admissions, were both valid and associated with adverse events subsequently evident during hospitalization.

We found strong evidence that SPICES is related to other measures of frailty and vulnerability. Direct assessment of the VES-13 scale among the sample of Medicare patients aged 65 or older was conducted at the same time as other SPICES criteria were documented. The correlation between the sum of SPICES and the VES-13 score was moderately high (Rho = 0.559). Sum of SPICES was also significantly associated with the Charlson Comorbidity Index, a measure of the burden of acute and chronic disease that is associated with risk of mortality. Age in years and a commonly used PHQ-2 screening tool for depression were also significantly related to the sum of SPICES criteria positive.

Frail hospitalized adults in this study were frequently unable to respond to the more complex VES-13 and PHQ-2 scales and might have been missed in screening if these scales were used to identify vulnerability. SPICES, on the other hand, can be scored for all inpatients and may, therefore, more completely screen acutely ill inpatients for frailty and/or vulnerability.

An additional strength of SPICES is that the screening elements are commonly assessed by bedside nurse in the routine admission assessment. The elements, recorded in documentation flow sheets in the electronic health record, could be electronically summarized and be used to trigger alerts or best practice guidelines.

In this sample of Medicare patients, individuals meeting two or more SPICES criteria had a risk of experiencing adverse events that were three times greater than individuals meeting none or one of these criteria. Less evident was the relationship of SPICES with specific adverse hospital outcomes. This finding may be explained, in part, by the observation that although 38.5% (67/174) of patients experienced at least one adverse event, the median incidence of any single events was 2.3%. A larger sample would be needed to establish the relative strengths of associations between SPICES criteria and specific adverse events. Individual risks may also be influenced by safety interventions and other factors that may change over time and differ among institutions. The main point that can be appreciated from these results is the potential utility of SPICES criteria for identifying a population at significant risk for adverse events within 24 hours of admission.

Patient care assessments are foundational in the determination of patient risks and the creation of effective interprofessional plans of care to assure the delivery of safe, quality, efficient patient care. Nursing admission assessments promote identification of patient risks and are important to the identification of patient care needs. Referrals to other patient care team members are part of the plan for patient care and essential to the achievement of positive patient outcomes. The finding of longer length of stay among patients with two or more SPICES positive in this study may be an effect associated with work flow and processes associated with hospital consultations and interventions requested to address and reduce risks for adverse outcomes, such as physical, occupational, and speech therapies, wound care, nutrition services, or social work. The SPICES may be a useful screening to trigger a comprehensive interprofessional care team response to further investigate patient risks and organize a prompt care plan response that might serve to both reduce length of stay and reduce the risk of adverse hospital outcomes.

### 3.5. Limitations

Patients were a convenience sample, not a random sample, so generalizability is limited. A larger study with a design that would ensure representativeness could add to the evidence. Scoring of SPICES from nursing documentations was limited by the single source of information on the element of sleep disturbance—admitting nurse's documentation of airway compromise. However, the absence of more complete documentation on this one element does not alter the findings, and one might expect that addition of a more complete assessment of sleep would only enhance the robustness of the SPICES score.

## 4. Conclusions

SPICES criteria constitute a valid assessment for screening for risks associated with adverse hospital outcomes. SPICES is strongly related to a screening assessment developed for community-residing older adults (VES-13) but more easily completed than the VES-13 by registered nurses as part of a hospital admission assessment. Most components of SPICES are already included in the admission nursing assessment and missing elements can easily be added. Use of SPICES as a screening protocol may serve to identify patient care needs for additional interventions by physicians, social workers, therapists, dieticians, pharmacists, and other members of the health care team.

## Figures and Tables

**Table 1:** Description of SPICES scoring criteria and numbers positive in the sample (*N* = 174).

SPICES element	Coding criteria	Number positive	Percent positive
Skin integrity	Documented presence of a pressure ulcer on admission (by a registered nurse and/or physician), wound consult ordered, or Braden risk score [8] of less than 16	72	41.4%

Problems eating	Katz Index of ADL [9]—self-feeding with partial or total help; patient identified with recent weight loss; admitting nurse made a referral for dietary consult; documented presence of a feeding tube upon admission; or patient assessed to have dysphagia at admission	79	45.4%

Incontinence	Katz Index of ADL bowel or bladder incontinence	69	39.7%

Confusion	Brief Interview for Mental Status (BIMS) questionnaire [10]; research nurse assessment of ability to answer questions; or altered mental status present on admission	73	42.0%

Evidence of falls	Admitting nurse's rating of the Morse Falls Risk Scale, score ≥45	117	67.2%

Sleep disturbance	Admitting nurse's screen for airway compromise	4	2.3%

**Table 2:** Distribution of total SPICES identified for patients (*N* = 174).

Number of SPICES+	Frequency	Percent	Cumulative percent
0	20	11.5	11.5
1	45	25.9	37.4
2	33	19.0	56.3
3	31	17.8	74.1
4	16	9.2	83.3
5	28	16.1	99.4
6	1	0.6	100.0

**Table 3:** Odds associated with positive frailty screen and selected validation measures (*N* = 174).

Validation measure	Odds ratio	95% Confidence interval	*P* value
Concurrent—criterion validity			
Vulnerable Elders Survey-13 frail >3 (*n* = 90)	11.077**	4.542, 27.013	<0.001
Charlson Comorbidity Index ≥2 (*n* = 125)	3.171**	1.600, 6.283	0.001
Charlson Comorbidity Index ≥4 (*n* = 63)	2.322**	1.176, 4.582	0.001

Concurrent—construct validity			
Age ≥ 80 years (*n* = 89)	4.345**	2.240, 8.430	<0.001
PHQ-2 screening ≥2 positive for depression (*n* = 55)	2.794**	1.364, 5.723	0.006
Admitted from a nursing home (*n* = 20)	1.235**	1.126, 1.355	<0.001
Prior hospital admission within 30 days (*n* = 48)	1.661	0.806, 3.382	0.220
2+ prior admissions within 6 months (*n* = 39)	1.253	0.592, 2.654	0.580
4+ active comorbid diagnoses, 1+ not controlled (*n* = 127)	2.794**	1.405, 5.557	0.004
≥7 prescription medications present on admission (*n* = 81)	2.916**	1.505, 5.649	0.004
Female gender (*n* = 102)	1.851	0.911, 3.456	0.058

Predictive validity			
Expired during admission (*n* = 3)	1.028	0.996, 1.061	0.294
Any hospital adverse event* (*n* = 67)	3.041**	1.527, 6.054	0.001
Readmission within 30 days (*n* = 38)	1.235	0.580, 2.627	0.705

*Adverse events summarized as one or more of the following: one or more falls (*n* = 5); hospital acquired pressure ulcer (*n* = 2); transfer to higher level of care (*n* = 10); complication from procedure (*n* = 9); any hospital acquired infection (*n* = 10); adverse drug reaction (*n* = 22); length of stay ≥7 days (*n* = 51). **indicates significant odds ratios.
